# Engine breakdown of lysosomes and related organelles and the resulting physiology

**DOI:** 10.3389/fcell.2025.1575571

**Published:** 2025-06-16

**Authors:** Nina Bakker, Marlieke L. M. Jongsma, Jacques Neefjes

**Affiliations:** Department of Cell and Chemical Biology and Oncode Institute, Leiden University Medical Center, Leiden, Netherlands

**Keywords:** lysosomes, lysosome-related organelles (LROs), transport, kinesin, dynein, microtubules, disease

## Abstract

Late endosomes/lysosomes (LE/Lys) and lysosome related organelles (LROs) move dynamically through cells which involves many levels of regulation. To reach their destination, they need to connect to the motor proteins dynein-dynactin, kinesin or myosin for long-range bidirectional transport along microtubules and short-range movement along actin filaments. This connection depends on various factors at the microtubule, including the MAP- and tubulin-code, as well as adaptors, Rab GTPases and effector proteins marking the LE/Lys and LRO membranes. Mutations affecting this transport results in defective LE/Lys or LRO cargo delivery often resulting in skin, neurological and/or immunological diseases. How LE/Lys and LRO transport is orchestrated and how it fails in disease states, will be discussed.

## Introduction

**FIGURE 1:**
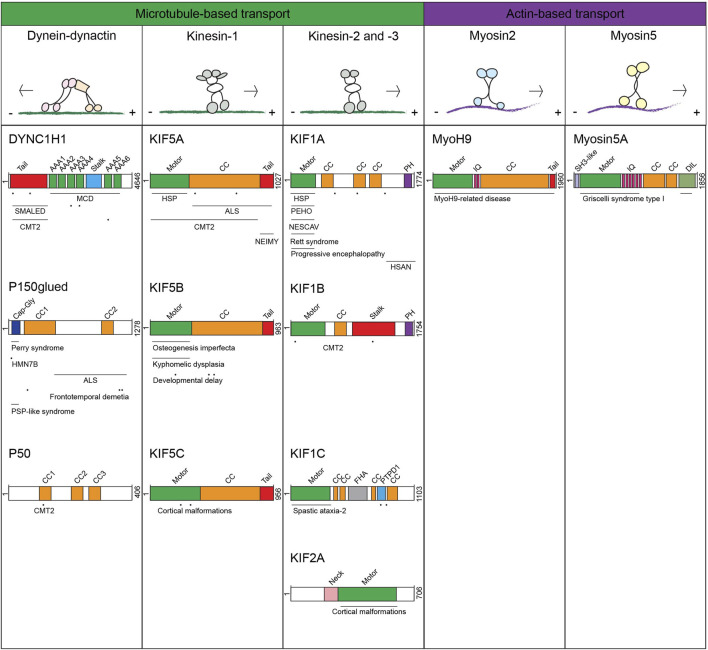
Overview of different motor proteins transporting late endosomes, lysosomes and lysosome-related organelles and mapping of mutations associated with motor protein-associated diseases Upper panel: motor proteins transporting late endosomes, lysosomes and lysosome-related organelles with their track and direction of movement. Lower panel: schematic representation of the protein domains and associated diseases per proteins. Horizontal lines above the mentioned disease indicate multiple mutations have been found in this (these) domain(s), dots indicate specific locations of mutations that have been reported.

Motor protein controlled vesicular transport is essential for maintaining cellular homeostasis. Without motor protein support, vesicles will not move in cells. Various types of vesicles are transported through the cellular space to deliver their cargo in response to signaling and cellular demand. These include compartments of the endolysosomal system (early endosomes and late endosome/lysosomes (LE/Lys)), Golgi-derived vesicles, ER-derived vesicles, peroxisomes, autophagosomes and lipid droplets. Furthermore, specialized cell types contain dedicated lysosome-related organelles (LROs) packed with specific cargo often destined for secretion, such as melanosomes in melanocytes, lytic granules (LGs) in cytotoxic T-cells (CTLs) and Natural Killer (NK)-cells and secretory vesicles in neurons (Delevoye et al., 2019; Marks et al., 2013; Raposo et al., 2007). To reach their destination, vesicles need to be actively transported. They can be transported in a fast, bidirectional manner along microtubules whereas short-range transport occurs along actin filaments. Microtubule-based transport is facilitated by two groups of motor proteins: the dynein-dynactin complex for minus-end directed (inward) movement, and the members of the kinesin (KIF) family moving cargo in the opposite direction (plus-end directed/outwards) (Figure 1) (Endow et al., 2010; Hook and Vallee, 2006; Bonifacino and Neefjes, 2017). The family of myosin motor proteins mediates transport along actin filaments (Bonifacino and Neefjes, 2017).

### The GTPases dance

Motor proteins require specific adaptors to attach to their cargoes. These adaptors usually involve small GTPases from the Rab, Arf and Arf-like (Arl)-family and interacting co-factors, adaptor and effector proteins at the target-membrane, or phosphoinositides (Stenmark, 2009; Donaldson and Honda, 2005; Balla, 2013; Li and Marlin, 2015; Posor et al., 2022). Approximately 60 different Rab and around 20 Arf/Arl small GTPases extensively label different organelles in mammalian cells (Pasqualato et al., 2002; Homma et al., 2021). Various lipids and/or small GTPases define the target membrane and thus the motor-type and the resulting transport. Small GTPases act as molecular switches that are activated when loaded with GTP and inactivated by GTP hydrolysis, a process accelerated by specific GAP proteins. Rab/Arf/Arl GTPases bind target vesicles in their activated GTP-bound state and then recruit effector proteins to mediate motor protein binding for cargo transport initiation. For example, LE/Lys are marked by Rab7, which recruits the effector proteins RILP or FYCO1 for dynein-dynactin or kinesin-motor dependent transport, respectively. The Rab7-positive LE/Lys can further mature into a LE/Lys marked by Arl8b. Arl8b provides a platform to recruit the GAP of Rab7 that then is removed. This yields a Rab7-Arl8b handover mechanism and illustrates how vesicle maturation is molecularly controlled (Jongsma et al., 2020). Arl8b recruits its own effectors, RUFY3/4 and JIP4 (recruiting dynein) or SKIP (recruiting kinesin), for bidirectional transport (Rosa-Ferreira and Munro, 2011; Keren-Kaplan et al., 2022; Kumar et al., 2022). Recently, the GEF DENND6A was shown to bind Arl8b, whereafter it activates another GTPase, Rab34, leading to recruitment of effector protein RILP and then the dynein-dynactin motor resulting in vesicle transport in the retrograde direction (Kumar et al., 2024). Thus multiple GTPases with different effectors binding oppositely directed motor proteins are recruited during the life- and maturation-time of LE/Lys.

LROs are like LE/Lys marked by different Rabs and effectors recruiting fusion machinery and motor proteins that are often uniquely expressed in the specialized cell types. For example, in melanocytes, melanin-containing melanosomes change their membrane composition during maturation. Melanosomes are then transported bidirectionally along microtubules by Rab36-RILP-Mreg-p150^glued^ complex formation resulting in dynein-dynactin mediated retrograde transport (Ohbayashi et al., 2012; Matsui et al., 2012) and Rab1A-SKIP-kinesin-1 (KIF5B) complex formation for anterograde transport (Ishida et al., 2015), albeit this is not confirmed by others (Robinson et al., 2017). At the end of the microtubules, melanosomes have to pass the cortical actin cytoskeleton under the cell surface to secrete their melanin content. Mature melanocytes acquire the small GTPase Rab27a that binds effector protein melanophilin and subsequently the actin-based motor Myosin5a (Wu et al., 2006; Park et al., 2019). As a result, the mature melanosomes accumulate below the cell membrane for secretion, which likely involves actin disassembly and formation of a functional SNARE complex (Van Den Bossche et al., 2006). The situation is similar in neurons and immune cells. NK-cells and CTLs contain LGs packed with cytotoxic enzymes that are released after activation in a more-or-less closed synapse with a target cell to-be-killed. LGs are transported towards the microtubule-organizing center (MTOC) (which reorientates towards the formed immune synapse) in a Rab7-RILP-dynein-dynactin dependent manner while Rab27a-Slp3-kinesin-1 (KIF5B) complex formation (in CTLs) as well as Arl8b-SKIP-kinesin-1 (KIF5B) complex formation (in NK-cells) mediates LGs anterograde transport (Tuli et al., 2013; Kurowska et al., 2012). The MTOC locates the LGs close to the membrane after activation of the NK-cell or CTL, allowing swift delivery of content (Daniele et al., 2011). Yet, the LGs have to pass the actin cytoskeleton involving the UNC45A-Myosin2a complex (Iizuka et al., 2015). Transport is more complicated in neurons, for the simple reason that microtubule-based transport goes along long distances to deliver the vesicles from the cell body to the axon terminus (Guedes-Dias and Holzbaur, 2019). Anterograde and retrograde transport is strictly coordinated in these axons by adaptor proteins that bind dynein-dynactin and kinesin-1 (KIF5B) motor proteins. For example, Alzheimer’s β-amyloid precursor protein (APP)-positive neuronal vesicles bind to JIP1 (Matsuda et al., 2001; Scheinfeld et al., 2002), which interacts with both dynein-dynactin and FEZ1-KIF5B depending on its phosphorylation state (Fu and Holzbaur, 2013; Blasius et al., 2007), while GABA(A)R-marked neuronal vesicles recruit HAP1-huntingtin (htt) to coordinate the activity of dynein-dynactin and kinesin-1 (KIF5B) motor proteins (Twelvetrees et al., 2010; Caviston et al., 2007; McGuire et al., 2006). With the long distances that need to be travelled in neurons, it is not surprising that mutations in protein complexes transporting these vesicles are usually first recognized in diseases with a neurological basis (see later). A list of diseases related to mutations in transport machineries is given in (Table 1).

**TABLE 1:** overview of transport-related proteins of which mutations are associated with disease.

Protein	Function	Associated diseases	References
APP	Adaptor protein	Alzheimer’s disease	Kang et al. (1987), Goate et al. (1991)
BICD2	Adaptor protein	Spinal muscular atrophy with lower extremity dominance	Oates et al. (2013), Peeters et al. (2013), Neveling et al. (2013)
BLOC1S3 (BLOS3)	BLOC1 complex	Hermansky-Pudlak syndrome 8	Morgan et al. (2006)
BLOC1S5 (muted)	BLOC1 complex	Hermansky-Pudlak syndrome 11	Pennamen et al. (2020)
BLOC1S6 (Pallidin)	BLOC1 complex	Hermansky-Pudlak syndrome 9	Badolato et al. (2012)
BLOC1S8 (dysbindin)	BLOC1 complex	Hermansky-Pudlak syndrome 7	Li et al. (2003)
		Schizophrenia	Straub et al. (2002)
CEP169	MT-associated	Autism	Chahrour et al. (2012)
CLN3	Rab7 interactor	Neuronal ceroid lipofuscinosis (Batten disease)	Isolation of a novel gene underlying Batten disease (1995)
CLN5	Rab7 interactor	Neuronal ceroid lipofuscinosis	Savukoski et al. (1998)
Dynein heavy chain	Dynein-dynactin motor	Spinal muscular atrophy with lower extremity dominance	Harms et al. (2012)
		Malformations in cortical development	Willemsen et al. (2012)
		Charcot Marie Tooth disease type 2	Weedon et al. (2011)
EB2	MT-associated	congenital symmetric circumferential skin creases-2	Isrie et al. (2015)
FYCO1	Adaptor protein	Cataract	Chen et al. (2011)
HPS1	BLOC3 complex/GEF	Hermansky-Pudlak syndrome 1	Fukai et al. (1995); Wildenberg et al. (1995); Oh et al. (1996)
HPS2/adaptin/AP3B1	AP3 complex	Hermansky-Pudlak syndrome 2	Dell'Angelica et al. (1999)
HPS3	BLOC2 complex	Hermansky-Pudlak syndrome 3	Anikster et al. (2001)
HPS4	BLOC3 complex	Hermansky-Pudlak syndrome 4	Suzuki et al. (2002)
Huntingtin	Adaptor protein	Huntington’s disease	Marcy et al. (1993)
JIP3	Adaptor protein	Neurodevelopmental disorder with or without variable brain abnormalities	Iwasawa et al. (2019)
KIF1A	Kinesin-3 motor	Hereditary Spastic Paraplegia	Erlich et al. (2011)
		Hereditary sensory and autonomic neuropathy	Riviere et al. (2011)
		NESCAV syndrome	Hamdan et al. (2011)
		PEHO syndrome	Langlois et al. (2016)
		Rett syndrome	Wang et al. (2019)
		progressive encephalopathy and brain atrophy	Esmaeeli et al. (2015)
KIF1B	Kinesin-3 motor	Charcot Marie Tooth disease type 2	Zhao et al. (2001)
KIF1C	Kinesin-3 motor	autosomal recessive spastic ataxia-2	Dor et al. (2014)
KIF2A	Kinesin-2 motor	Cortical malformations	Poirier et al. (2013)
KIF5A	Kinesin-1 motor	Hereditary Spastic Paraplegia	Reid et al. (2002)
		Amyotrophic Lateral Sclerosis	Nicolas et al. (2018)
		Charcot Marie Tooth disease type 2	Crimella et al. (2012); Brenner et al. (2018)
		Neonatal Intractable MYoclonus	Duis et al. (2016)
KIF5B	Kinesin-1 motor	Osteogenesis imperfecta	Marom et al. (2023)
		kyphomelic dysplasia	Itai et al. (2022)
		Developmental delay with variable symptoms including myopathic features	Flex et al. (2023)
KIF5C	Kinesin-1 motor	Cortical malformations	Poirier et al. (2013)
LIS1	MT-associated	Lissencephaly	Reiner et al. (1993)
LYST	Adaptor protein	Chediak-Higashi syndrome	Barbosa et al. (1996)
MAP6	MT-associated	schizophrenia	Merenlender-Wagner et al. (2014); Shimizu et al. (2006)
MID1	MT-associated	Opitz syndrome	Quaderi et al. (1997)
MLPH	Myosin effector	Griscelli syndrome type 3	Menasche et al. (2003)
MyoH9	Myosin motor	MYOH9-related disease	Kunishima et al. (1999); Seri et al. (2000)
Myosin 5a	Myosin motor	Griscelli syndrome type 1	Pastural et al. (1997)
Myosin 7a	Myosin motor	Usher syndrome type 1B	Weil et al. (1995)
NPC-1	Cholesterol transporter	Niemann Pick Type C1-disease	Carstea et al. (1997)
NPC-2	Cholesterol transporter	Niemann Pick Type C2-disease	Naureckiene et al. (2000)
P50/dynamitin	Dynein/dynactin motor	Charcot Marie Tooth disease type 2	Braathen et al. (2016)
P150glued	Dynein-dynactin motor	Perry syndrome	Farrer et al. (2009)
		hereditary motor neuronopathy 7B	Puls et al. (2003)
		Frontotemporal dementia	Munch et al. (2005)
		progressive supranuclear palsy-like syndrome	Stockmann et al. (2013)
		Amyotrophic Lateral Sclerosis	Munch et al. (2004)
Rab7a	Small GTPase	Alzheimer’s disease	Vardarajan et al. (2012)
		Charcot Marie Tooth disease type 2	Verhoeven et al. (2003)
RAB27a	Small GTPase	Griscelli syndrome type 2	Menasche et al. (2000)
Rab32	GEF	Parkinsons	Gustavsson et al. (2024); Hop et al. (2024)
SNX1	Retromer complex	Alzheimer’s disease	Vardarajan et al. (2012)
Tau	MT-associated	Alzheimer’s disease	Grundke-Iqbal et al. (1986); Conrad et al. (2002)
		Frontotemporal dementia	Joachim et al. (1987); Hutton et al. (1998)
		progressive supranuclear palsy	Bancher et al. (1987); Conrad et al. (1997))
		Parkinson’s disease	Joachim et al. (1987); Martin et al. (2001)
VAPB	MCS	Amyotrophic Lateral Sclerosis	Nishimura et al. (2004)
VPS11	HOPS complex	Hypomyelinating Leukodystrophy 12	Edvardson et al. (2015)
		Dystonia 32	Monfrini et al. (2021)
VPS16	HOPS complex	Dystonia 30	Cai et al. (2016); Steel et al. (2020))
VPS33a	HOPS complex	mucopolysaccharidosis-plus syndrome	Dursun et al. (2017)
VPS35	Retromer complex	Parkinson’s disease	Vilarino-Guell et al. (2011); Zimprich et al. (2011)
VPS39	HOPS complex	Schizophrenia	Xu et al. (2012)
VPS41	HOPS complex	autosomal recessive spinocerebellar ataxia-29	Steel et al. (2020)

### The microtubule highway and traffic control

Fast vesicle transport occurs along microtubules by microtubule-based motor proteins. These microtubules are not empty roads but covered by various microtubule-associated proteins (MAPs). The MAP-family consists of different proteins including microtubule stabilizing proteins and proteins controlling motor protein transport (Jijumon et al., 2022; Bodakuntla et al., 2019; Goodson and Jonasson, 2018; Matteoni and Kreis, 1987) (summarized in (Table 2)). MAP2, MAP4, MAP7 and MAP9 can inhibit or activate dynein-dynactin and kinesin motor proteins (Jongsma et al., 2023; Monroy et al., 2020; Monroy et al., 2018; Hagiwara et al., 1994; Paschal et al., 1989; Hooikaas et al., 2019; Chaudhary et al., 2019; Barlan et al., 2013; Semenova et al., 2014; Ferro et al., 2022), while other MAPs are involved in motor protein recruitment to the microtubule. These include the atypical dynein heavy chain (dynein HC) activating MAP LIS1, as well as the E3 ligase MID1 and the plus-end proteins EB1 and CEP169 that recruit activated dynein HC to the microtubule growing plus-end (Splinter et al., 2012; Elshenawy et al., 2020; Singh et al., 2024; Gillies et al., 2022; Baumbach et al., 2017; Karasmanis et al., 2023; Dixit et al., 2008a; Duellberg et al., 2014; Jongsma et al., 2024). The dynein HC has to assemble with the dynactin complex to form a functional dynein-dynactin motor, which is recruited to the microtubule plus end by factors including EB1 and CLIP170 (Jongsma et al., 2024; Bjelic et al., 2012; Watson and Stephens, 2006). Consequently, the dynein-dynactin motor is assembled at the plus-end and then moves inward to catch associated LE/Lys for minus end transport. LRO transport is also regulated by MAPs. For example, MAP4 mediates switching between kinesin and dynein-dynactin dependent transport in melanocytes. When dephosphorylated, MAP4 binds to the microtubule surface where it recruits kinesin-2 towards the melanosome, whereas phosphorylated MAP4 is cytosolic and unable to recruit kinesin-2, thereby favoring dynein-mediated transport (Semenova et al., 2014). The neuronal MAP tau stabilizes and bundles axonal microtubules (Chung et al., 2016). In addition, tau inhibits kinesin-1 and kinesin-3 microtubule binding and motility without effecting kinesin-2 and dynein-mediated transport of LE/Lys and LROs (Monroy et al., 2020; Monroy et al., 2018; Chaudhary et al., 2018). Yet, the control of cargo transport along microtubules is even more complicated by various tubulin isotypes and post-translational modifications that also control microtubule stability and motor protein activation (McKenna et al., 2023). On these modification and protein littered roads, vesicles and their associated microtubule-based motor proteins have to find their ways while moving in a bidirectional and stop-and-go manner.

**TABLE 2:** Overview of microtubule-associated proteins regulating motor proteins and microtubule dynamics.

MAP	Regulation of motor proteins	Function in MT dynamics
Tau	Obstruction of kinesin-1 (KIF5B) and kinesin-3 (KIF1A), without affecting dynein Monroy et al. (2018); Chaudhary et al. (2018); Dixit et al. (2008b); Tan et al. (2019); Vershinin et al. (2007); McVicker et al. (2011)	MT stabilization and bundling Chung et al. (2016)
MAP6		MT coiling and stabilization Cuveillier et al. (2020)
EB1	Dynein and dynactin loading on plus-end Baumbach et al. (2017); Duellberg et al. (2014); Jongsma et al. (2024); Moughamian et al. (2013)	Recruitment of plus-end binding proteins, regulating plus-end dynamics, MT growth, minus-end organization Bieling et al. (2008); Maurer et al. (2014); Yang et al. (2017); Maurer et al. (2012); Bieling et al. (2007); Akhmanova and Steinmetz (2008)
EB2		Regulating plus-end dynamics, MT destabilization Maurer et al. (2012); Akhmanova and Steinmetz (2008); Zhong et al. (2021)
EB3		Regulating plus-end dynamics, MT growth, minus-end organization Yang et al. (2017); Maurer et al. (2012); Akhmanova and Steinmetz (2008); Nakagawa et al. (2000)
MID1	Dynein loading on plus-end Jongsma et al. (2024)	MT Stabilization Schweiger et al. (1999); Berti et al. (2004)
LIS1	Dynein activation and loading on plus-end Splinter et al. (2012); Elshenawy et al. (2020); Singh et al. (2024); Gillies et al. (2022); Baumbach et al. (2017); Karasmanis et al. (2023); Jongsma et al. (2024)	
CEP169	Dynein loading on plus-end Jongsma et al. (2024)	MT stabilization and acetylation Mori et al. (2015b); Mori et al. (2015c)
MAP4	Positive modulation of kinesin-2 while inhibiting kinesin-1 and dynein-dynactin Semenova et al. (2014); Tokuraku et al. (2007)	MT assembly and mitotic spindle orientation Aizawa et al. (1991); Samora et al. (2011); Permana et al. (2005)
MAP2	Inhibition of Kinesin-1 (KIF5B), Kinesin-3 (KIF1A) and Dynein-dynactin; inhibition of kinesin-1 while allowing kinesin-3 from the soma into the axon Monroy et al. (2020); Hagiwara et al. (1994); Paschal et al. (1989); Gumy et al. (2017)	MT stabilization and rigidity Felgner et al. (1997); Itoh et al. (1997); Dye et al. (1993)
MAP7	Positive modulation of Kinesin-1 (KIF5B) Monroy et al. (2020); Monroy et al. (2018); Hooikaas et al. (2019); Chaudhary et al. (2019); Ferro et al. (2022)	MT Stabilization Masson and Kreis (1993); Tymanskyj and Ma (2019)
MAP7D1	Positive modulation of Kinesin-1 (KIF5B) Hooikaas et al. (2019)	Maintenance of acetylated MTs Kikuchi et al. (2022)
MAP7D2	Positive modulation of Kinesin-1 (KIF5A/B/C) Hooikaas et al. (2019)	MT stabilization Kikuchi et al. (2022)
MAP7D3	Positive modulation of Kinesin-1 (KIF5B) Hooikaas et al. (2019)	MT stabilization and assembly Yadav et al. (2014); Sun et al. (2011); Tala et al. (2014)
MAP9	Positive modulation of Kinesin-3 (KIF1A) Monroy et al. (2020)	MT stabilization and spindle assembly Saffin et al. (2005); Wang et al. (2020)

Although microtubules allow transport over long distances in cells, the microtubule highways fail to deliver cargo to the plasma membrane, as they do not reach the cell surface. Where microtubules end, the cortical actin network takes over. To move vesicles along actin cables requires a different set of motor proteins, the myosin motor proteins. At the growing plus-end of microtubules, the EB1 protein can orchestrate the hand-over of melanosomes from microtubule-based motors to the actin-associated motor Myosin5a, which is an important step preceding plasma membrane fusion and release of melanin. EB1 binds Melanophilin-Myosin5a allowing transfer of the Melanophilin-Myosin5a complex to GTPase Rab27a as present at the mature melanosomes membrane, initiating actin-binding before membrane fusion (Wu et al., 2005). Similarly, Myosin5a facilitates the delivery of neuronal vesicles (Lise et al., 2006; Takamori et al., 2006; Correia et al., 2008; Roder et al., 2010; Roder et al., 2008), whereas LGs are transported by UNC45-Myosin2 (Iizuka et al., 2015; Krzewski et al., 2006). When the plasma membrane is reached, this activates SNARE-complex assembly and fusion. The road to plasma membrane delivery is complicated and dynamic but it works!

LE/Lys and LRO transport is controlled by a series of molecules acting at different levels. It is therefore not too surprising that mutations in the different proteins involved can result in disease states. We will dissect the different steps in this pathway and describe the different mutations found in the mammalian system and their phenotypes. We will start at the microtubules and then work our way up to the LE/Lys and LROs by discussing the motor proteins and adaptor/effector proteins bound at the various cargo membranes. In assembly, they illustrate the relevance of proper control of lysosomal transport processes for a healthy state.

## At the microtubule highways

Intracellular long-range motor-mediated transport is possible through a functional highway-system, the microtubule network, build-up from various tubulin subunits. These microtubules, their modifications and their associated proteins are in control of LE/Lys and LRO transport.

### Diseases associated with microtubules and their associated proteins

The basis of microtubules is formed by different isotypes of α- and β-tubulin dimers. Missense and splice-site mutations in genes encoding for tubulin subunits result in various diseases including lissencephaly brain and ocular cranial nerve disorders, illustrating the importance for proper maintenance of these trafficking-roads (Tischfield et al., 2011). Besides the tubulin isotypes, there are many microtubule-associated post-translational modifications including acetylation, ubiquitination, sumoylation, detyrosination, glutamylation and phosphorylation (McKenna et al., 2023). This so-called tubulin-code controls microtubule dynamics and stability, can act at the growing plus-end and controls vesicle transport and issues like neuronal growth, differentiation and axonal regeneration (Lu et al., 2024). The various enzymes involved in the different post-translational modifications are then expected to yield neuronal diseases when mutated. Indeed, mutations in DYRK1A, which phosphorylates Ser172 on β -tubulin, have been associated with intellectual developmental disorder (van Bon et al., 2011; O'Roak et al., 2012; Courcet et al., 2012). However, as DYRK1A is involved in the phosphorylation of many targets, including transcription factors such as CREB, FKHR, GLI1, NFAT, and STAT3, it is unlikely that the disease is solely caused by the loss of β-tubulin phosphorylation. Mutations in the tubulin-glycosylating enzyme TTLL10 have been identified in patients with severe bleeding disorder, where it is suggested to play a crucial role in the microtubule dynamics involved in platelet production (Khan et al., 2022). In addition to the tubulin PTMs, there are many MAPs decorating the tubulin subunits, together forming the MAP-code (Monroy et al., 2020), ochestrating many different functions, including the control of dynein and kinesin motor protein-mediated transport. A well known MAP affecting transport is tau, encoded by the *MAPT* gene, which is specifically expressed in neuronal cells. Aggregates containing hyperphosphorylated Tau are linked to multiple neurodegenerative diseases including Alzheimer’s Disease (Grundke-Iqbal et al., 1986). In some studies, certain MAPT variants associated with an increased risk of tauopathies, likely due to increased MAPT expression (Tanahashi et al., 2004; Laws et al., 2007; Myers et al., 2007; Russ et al., 2001; Baker et al., 2000; Bullido et al., 2000; Clark et al., 2003; Kwok et al., 2004). When binding the microtubule surface, tau obstructs vesicle transport by acting as an obstacle for kinesin and dynein motor proteins (Vershinin et al., 2008; Ebneth et al., 1998), which can then induce neurodegeneration (Chaudhary et al., 2018). Another neuronal specific MAP suggested to be associated to disease is MAP6. MAP6 mutant mice are a model for schizophrenia and show a reduced presynaptic glutamate vesicle density (Andrieux et al., 2002; Daoust et al., 2014; Gimenez et al., 2017; Merenlender-Wagner et al., 2014). Furthermore, MAP6 expression was upregulated in post-mortem brains of schizophrenia patients, along with two SNPs that showed an association with schizophrenia (Shimizu et al., 2006).

At the microtubule plus-end, various MAPs regulate the dynamic growth and shrinkage (catastrophy) of the microtubule. This process is mainly regulated by the end-binding family proteins, EB1-3. EB1, MID1 and CEP169 (encoded by *MAPRE1*, *MID1* and *NCKAP5L* genes) are essential to locate the activated (Lis1 containing) dynein HC at the growing microtubule plus-end (Jongsma et al., 2024). So far, altered expression of EB1 has been observed in pediatric ependymoma (underexpression) (Suarez-Merino et al., 2005) and esophageal squamous cell carcinoma (overexpression) (Wang et al., 2005), as well as a lymphoblastic leukemia patient showing a fusion of EB1 and MLL (Mixed-Lineage Leukemia) (Fu et al., 2005). Mutations in the *MID1* gene have been linked to Opitz Syndrome, a disease caused by defects in cell migration resulting in maldeveloped midline structures (Schweiger and Schneider, 2003; Aranda-Orgilles et al., 2008). MID1 is known to stabilize microtubules (Schweiger et al., 1999), yet MID1 can also ubiquitinate phosphatase 2A (PP2A) leading to its degradation (Trockenbacher et al., 2001). Mutated MID1 therefore leads to increased PP2A levels which alters cytoskeletal remodeling, intracellular transport and cell migration (Perea-Cabrera et al., 2023). Next, dysfunctional centrosomal protein CEP169 has been associated to Autism spectrum disorder (ASD) (Chahrour et al., 2012). How mutations in CEP169 contribute to ASD is currently not understood, but it might be related to its function in synaptic plasticity, as CEP169 was found to be upregulated in response to neuronal activity (Chahrour et al., 2012), similarly to other genes associated with autism (Walsh et al., 2008; Ramocki and Zoghbi, 2008).

The MAP-code controls both the microtubule highway and the activation of kinesin and dynein-dynactin motor proteins. It is therefore not surprising that many diseases associated to LE/Lys and LRO transport are the result of mutations in these proteins. How about the motor proteins, which need to walk along these roads?

## Dynein-Dynactin mediated transport

Dynein-dynactin is a large, multi-subunit motor protein complex interacting with cargo adaptors and effector proteins to transport a various cargoes towards the microtubule minus-end, including vesicles, mitochondria and mRNA (Wilkie and Davis, 2001; Pilling et al., 2006; Jordens et al., 2001; Gross et al., 2002; Driskell et al., 2007). It consists of two multi-subunit complexes, the dynein motor and its cofactor dynactin, which assemble as an active motor at the microtubule plus-end after recruitment by various MAPs (Jongsma et al., 2024; Bjelic et al., 2012; Watson and Stephens, 2006). Dynein is formed by two dynein heavy chains (HCs) (*DYNC1H1*) activated by Lis1 (Elshenawy et al., 2020; Htet et al., 2020), an intermediate chain (IC, *DYNC1I1* or *DYNC1I2*), a light-intermediate chain (LIC, *DYNC1LI1* or *DYNC1LI2*), and three light chains (LCs, *DYNLT1*, *DYNLL1* and *DYNLRB1*) (recently reviewed by (Rao and Gennerich, 2024). The dynactin complex contains 23 proteins, including the microtubule binding subunit p150^glued^, built around a short filament of actin related protein-1 (Arp1) (Urnavicius et al., 2015). At the microtubule plus-end, two dynein dimers can assemble with one dynactin complex and (when available) a cargo adaptor (Splinter et al., 2012; Urnavicius et al., 2015; Schlager et al., 2014; McKenney et al., 2014). Since cells express just a single type of dynein HC motor that facilitates minus-end directed transport in the cytoplasm (Roberts et al., 2013), the formation with various IC, LIC and cargo adaptor proteins allows the specificity to regulate transport of distinct cargoes. Dynactin enhances the processivity of the dynein motor (King and Schroer, 2000). Mutations in the dynein-dynactin subunits should then affect transport of lysosomes and related organelles translating in disease phenotypes.

### Diseases related to defects in the dynein motor

Mutations in the human *DYNC1H1* gene (encoding the 500 kDa dynein HC) have been associated to multiple neurological diseases (Marzo et al., 2019; Hoang et al., 2017; Becker et al., 2020) (an overview of the motor proteins with associated diseases and location of mutations can be found in (Figure 1)). More than 40 heterozygous missense mutations in the *DYNC1H1* gene are identified in patients with malformations in cortical development (MCD, mutations mostly found in dynein HC motor-domain) and spinal muscular atrophy with lower extremity dominance (SMALED, mutations found in dynein HC tail-domain) (Becker et al., 2020; Willemsen et al., 2012; Weedon et al., 2011; Vissers et al., 2010; Tsurusaki et al., 2012; Strickland et al., 2015; Scoto et al., 2015; Schiavo et al., 2013; Poirier et al., 2013; Niu et al., 2015; Mei et al., 2023; Lipka et al., 2013; Harms et al., 2012; Gelineau-Morel et al., 2016; Fiorillo et al., 2014). These are neuromuscular disorders caused by defects in neuronal proliferation and migration resulting in intellectual disabilities and epilepsy (MCD) or spinal cord motor neuron loss affecting lower limp function (SMALED). A study investigating the effect of 14 MCD or SMALED-associated dynein HC mutations on dynein-dynactin function showed that most human disease-associated mutations reduced dynein-dynactin-BICD2 complex motility (Hoang et al., 2017). Peripheral neurons with long axons, requiring long distance transport, are likely the first cells affected by the reduced processivity of the dynein-dynactin-cargo complex, explaining why specifically lower extremities of the body are affected in SMALED. Stronger effects on dynein-dynactin motility also hamper retrograde transport in neurons with shorter axons, which contributes to the cortical malformations found in MCD. Another neurological disease is caused by mutations in the dynein HC tail-domain, essential for dynein HC dimerization, and is called Charcot Marie Tooth disease type 2 (CMT2), a progressive disease characterized by muscle weakness (Weedon et al., 2011). A mouse model mimicking human CMT2-associated mutations showed reduced innervation and lower synaptic vesicle density at the gastrocnemius neuromuscular junctions, likely caused by defective dynein transport of the synaptic vesicles (Weedon et al., 2011; Nandini et al., 2019).

### Diseases related to defects in the dynactin complex

Although consisting of 23 subunits, most dynactin mutations associated to neurodegenerative diseases are observed in the *DCTN1* gene encoding for the dynactin subunit p150^glued^. Mutations localizing to the CAP-gly domain in or close to the GKNDG-motif, which controls p150^glued^ binding to microtubules, have been identified in Perry syndrome and hereditary motor neuronopathy 7B (HMN7B) patients (Waterman-Storer et al., 1995; Schroer, 2004; Li et al., 2002). Perry syndrome is a neurodegenerative disease characterized by parkinsonism, psychiatric symptoms and TDP-43 (transactive-response DNA-binding protein of 43 kDa) aggregation in the brain. Perry syndrome-associated p150^glued^ mutants are able to dimerize and associate to the dynein IC and did not affect axonal transport, yet dynactin recruitment to microtubules is limited as is the inability to accumulate dynactin at neurite tips (Moughamian and Holzbaur, 2012). Although these P150 mutations only mildly affect microtubule binding, even such subtle effects on dynein motor transport can result in disrupted lysosome and autophagosome retrograde transport leading to accumulation and aggregation of pathological proteins (Perlson et al., 2010), including TDP-43 (Xia et al., 2016). But not all mutations in the p150^glued^ CAP-Gly domain lead to similar phenotypes. The Gly59Ser substitution in p150^glued^ has been diagnosed in just a few families as HMN7B disease (Puls et al., 2003). Whereas there is no evidence for motor neuron pathology in Perry syndrome, HMN7B patients show chronic motor neuron denervation and have an earlier onset of disease. The HMN7B Gly59Ser mutation is located at the center of the CAP-Gly domain while Perry Syndrome-related mutations are located at the protein surface (Moughamian and Holzbaur, 2012). This mutation is not directly involved in p150^glued^ binding to the microtubule, but since Serine-residues are greater in size than Glycine-residues the substitution will cause steric hindrance and disturb proper folding of the CAP-Gly domain resulting in mildly affected microtubule binding. This mutation not only decreases the interaction with dynein HC but also hinders dynactin recruitment to the microtubule resulting in reduced minus-end directed cargo transport. Of note, mutant p150^glued^ can form toxic aggregates that also contribute to death of neurons (Moughamian and Holzbaur, 2012). In addition, some rare mutations in the *DCTN1* gene have been associated to other neurodegenerative diseases, such as frontotemporal dementia, progressive supranuclear palsy-like syndrome and the motor neuron disease Amyotrophic Lateral Sclerosis (ALS), which results in loss of muscle control (Konno et al., 2017). Various p150^glued^ mutations are found in ALS patients (Met571Thr, Arg785Trp, Arg1101Lys and Thr1249Ile) that reside outside the CAP-Gly domain and do not affect microtubule binding and do not result in p150^glued^ aggregation (Dixit et al., 2008a; Stockmann et al., 2013). They affect p150^glued^ binding to the dynein HC motor (Munch et al., 2004). These mutations may form a genomic risk factor for developing ALS. However, as ALS is a multifactorial disease caused by a combination of mutations and environmental factors, ALS patients with p150^glued^ mutations should also include other ALS-associated genes (Cady et al., 2015), as not all family members carrying these *DCTN1* gene mutations developed ALS. Mutations in the p50/dynamitin subunit have been identified in patients with CMT2 (Braathen et al., 2016). The His113Tyr mutation is located in the first coiled-coil motif, which is predicted to mediate self‐association and stabilization of the dynactin complex (Jacquot et al., 2010; Maier et al., 2008). Mutations in other dynactin subunits are more rare but do occur.

### Diseases related to defects in dynein-dynactin activating adaptor proteins

The dynein-dynactin motor does not function alone, there are multiple dynein-activating adaptor proteins, including BICD-family, Hook-family and JIP-family proteins that bind the dynein-dynactin complex to enhance motor-complex stability and allow the motor to connect to specific cargo (Olenick and Holzbaur, 2019). Activating adaptors contain a N-terminal dynein-dynactin binding domain which often includes a domain interacting with the dynein LIC while its C-terminus is involved in cargo-binding (Reck-Peterson et al., 2018). Most adaptor proteins enhance dynein-dynactin motility, yet some, like JIP-family adaptors, TRAK1/2 and HAP1, act as motility switches by coordinating both dynein-dynactin and kinesin motors (Fu and Holzbaur, 2013; Twelvetrees et al., 2010; McGuire et al., 2006; Engelender et al., 1997; Li et al., 1998; van Spronsen et al., 2013). Some neuronal diseases have been associated to mutations in these adaptor proteins. For example, mutations in the *BICD2* gene are associated with SMALED (Fiorillo et al., 2016; Oates et al., 2013; Peeters et al., 2013; Picher-Martel et al., 2020; Ravenscroft et al., 2016), while a study in *C. Elegans* showed that mutations in the neuronal expressed *MAPK8IP3* gene (encoding the homologue of mammalian JIP3) results in disturbed LE/Lys trafficking associated to NEDBA (Neurodevelopmental Disorder with or Without Variable Brain Abnormalities) (Arimoto et al., 2011; Edwards et al., 2013). Also, mutations in the dynein activator *LIS1* gene lead to the neuronal migration disease Lissencephaly causing severe brain malformations (Dobyns et al., 1993; Guerrini and Parrini, 2010). Possibly, dysfunctional Lis1 affects neuronal migration as a result of defective nuclear movement. During neuronal migration, the nucleus couples to the centrosome, a process depending on dynein-mediated nuclear translocation. Depletion of LIS1 was found to hamper nucleus-centrosome coupling, which could be rescued by overexpression of wild-type Lis1 but not Lis1 constructs harboring Lissencephaly-associated patient mutations (Tanaka et al., 2004). Similarly, defects in neuronal migration are also observed when the dynein motor is inactivated, suggesting that Lis1 is required to activate the dynein motor for correct nuclear translocation (Tanaka et al., 2004).

It may be surprising that mutations with relatively small effects on dynein-dynactin motor transport already result in various neurological diseases. However, full inhibition of dynein motor activity will be lethal and it is likely that small effects are tolerated at the cost of diseases in the system most sensitive to alteration in microtubule based transport, the neurological system.

## The other direction: kinesin-mediated transport

The kinesin-superfamily (KIFs) consists of 14 subfamilies, which includes 45 different kinesin HCs (Lawrence et al., 2004; Hirokawa et al., 2009). In general, kinesins contain an N-terminal motor domain, followed by a family-specific neck region that determines the generated force, a coiled-coil domain for dimerization and a C-terminal tail defining cargo specificity. Two kinesin HCs form a dimer and assemble with various co-factors, including light chains for kinesin-1, allowing interaction with specific cargoes (Verhey and Hammond, 2009; Dimitrova-Paternoga et al., 2021). To become an active motor, kinesins require MAPs or other co-factors to be released from their autoinhibited state, as shown for MAP7-family members activating kinesin-1 member KIF5B (Hooikaas et al., 2019), and Kinesin-associated protein 3 (KAP3) activating the kinesin-2 heterodimers KIF3A–KIF3B and KIF3A–KIF3C by forming active heterotrimeric complexes (Sonar et al., 2020; Cole et al., 1993; Garbouchian et al., 2022; Yamazaki et al., 1995), thus supporting microtubule binding and subsequent cargo transport by these kinesin motors towards the microtubule plus-end.

### Diseases related to defects in the kinesin-motors

The kinesin motors reported to transport LE/Lys and most LROs are KIF1A/B (kinesin-3) and KIF5A/B/C (kinesin-1). While KIF1B and KIF5B are ubiquitously expressed, KIF1A, KIF5A and KIF5C are mainly expressed in neuronal cells (Niclas et al., 1994; Nangaku et al., 1994; Kanai et al., 2000; Aizawa et al., 1992). Of these, especially mutations in kinesin-1 family member KIF5A have been associated with neuronal diseases (Cozzi et al., 2024). The type of disease depends on the location of the KIF5A mutation. Hereditary Spastic Paraplegia (HSP) is caused by mutations in the KIF5A N-terminal motor domain. HSP-associated Arg17Gln and Arg280Cys mutant KIF5A showed reduced motility and microtubule binding capacity and destabilized the protein (Cozzi et al., 2024; Goizet et al., 2009; Ebbing et al., 2008; Fichera et al., 2004; Liu et al., 2014; Reid et al., 2002; Blair et al., 2006; Crimella et al., 2012). CMT2 is linked to KIF5A mutations in both the N-terminal motor domain and stalk region leading to truncated motor proteins without tail-domain (Cozzi et al., 2024; Crimella et al., 2012). ALS is associated to mutations in the KIF5A C-terminal cargo-binding tail disturbing cargo-binding and then cargo localization (Cozzi et al., 2024; Nicolas et al., 2018; Brenner et al., 2018). Another C-terminal KIF5A mutation, Cys975Valfs*73, elongates the KIF5A tail-domain. The extended tail region reduces solubility of the protein that then aggregates. This also reduces the number of active KIF5A motors, causing NEonatal Intractable MYoclonus (NEIMY) (Cozzi et al., 2024; Rydzanicz et al., 2017). The wide variety of diseases linked to KIF5A shows the importance of KIF5A-mediated transport in neuronal cells. However, also mutations in KIF1A (kinesin-3) and KIF5C (kinesin-1) yield defects in brain development leading to brain malformation (Poirier et al., 2013; Esmaeeli et al., 2015; Klebe et al., 2012; Lee et al., 2015; Michels et al., 2017; Riviere et al., 2011) as are mutations in KIF1B (kinesin-3), which have been linked to CMT2 (Zhao et al., 2001). The identified mutations in KIF1A, KIF1B and KIF5C cluster in the motor domains and result in reduced ATP hydrolysis capacity and consequently, reduced motility of the motors (Esmaeeli et al., 2015; Zhao et al., 2001). Mutations in the motor domain of KIF5B have also been observed, and are associated with skeletal dysplasias (Itai et al., 2022; Marom et al., 2023). The KIF5B Leu498Pro and Leu537Pro mutations were found in patients with developmental delay translating in variable symptoms including myopathic features, and localize to the KIF5B coiled-coil domains and likely affects KIF5B dimerization (Flex et al., 2023).

Since LE/Lys and LROs move along microtubules in a bidirectional manner involving dynein-dynactin as well as kinesin motor proteins, it would have been surprising if only one of these motors would associate to neurological diseases. Indeed, mutations in both motor classes are ultimately involved in a plethora of neurological disorders as they are active in the same process; long distance transport of LE/Lys and LROs. But there is a third class of motor proteins using another highway, the actin-based myosin motos. What about these?

## Myosin-mediated transport along actin highways

When LE/Lys and LROs move to the cell surface along microtubules, they will reach the actin cytoskeleton. Also this transport is polarized and requires myosin motors to move towards the actin plus-end, with the exception of Myosin6 that moves in the opposite direction (Wells et al., 1999). The Myosin-family can be divided into at least 20 subclasses (Odronitz and Kollmar, 2007; Sebe-Pedros et al., 2014; Richards and Cavalier-Smith, 2005; Foth et al., 2006), that participate in various trafficking and anchoring events at many cellular locations. Specificity of cargo binding occurs through the divergent myosin tail-regions while their N-terminal catalytic-domains are conserved between subclasses (Thompson and Langford, 2002).

### Diseases related to defects in myosin-motors

Since the last, but equally essential part in vesicle transport towards the plasma membrane involves myosin motors that control the actin based transport step, it is predictable malfunction at this transport step should also yield disease. Since multiple myosin motors (Myosin1 (Donaudy et al., 2003; Zadro et al., 2009), Myosin2 (Kunishima and Saito, 2010), Myosin3 (Walsh et al., 2002), Myosin6 (Melchionda et al., 2001), Myosin7 (Liu et al., 1997) and Myosin15 (Wang et al., 1998)) contribute to the structure of stereocilia essential for hearing (Nambiar et al., 2010), the most common abnormality related to mutations/dysfunction of these motor proteins is deafness. This is illustrated by Myosin7-related Usher syndrome (Kremer et al., 2006). Mutations in myosin motors are also associated to many other diseases, including the LRO-transport related diseases Griscelli syndrome Type 1 (GS1) and MYH9 (Myosin9)-related disease (Van Gele et al., 2009). GS1 is caused by mutations in the *MYO5A* gene, encoding Myosin5 HC, the motor that links Rab27A-marked melanosomes to actin filaments preceding plasma membrane fusion for melanin release. Similarly, it is involved in the release of LROs in neuronal cells (Lise et al., 2006; Takamori et al., 2006; Correia et al., 2008; Roder et al., 2010; Roder et al., 2008). Because of its function in both melanocytes and neurons, GS1 patients suffer from neurological abnormalities next to the well described pigmentation abnormalities. *MYO5A* mutations identified in GS1 patients include mutations in the motor domain, among which the nonsense mutation Arg779X resulting in truncated Myosin5 lacking its calmodulin, neck and tail region leading to complete loss-of-function (Pastural et al., 1997). In other patients, a 47 base pair insertion was identified at the start of the Myosin5 tail-domain leading to a truncated Myosin5 lacking its cargo binding tail (Pastural et al., 2000). Depending on cell type, *MYO5A* transcripts are alternatively spliced leading to various Myosin5 isoforms. While brain cells only express a shorter Myosin5 isoform, melanocytes mostly express Myosin5 with a longer tail domain (including the F-exon) essential for binding to melanophillin. Therefore, GS1 patients with F-exon deletions display pigmentation abnormalities while neuronal cells and function are unaffected (Menasche et al., 2003). MYH9-RD is a collection of disorders caused by mutations in the *MYH9* gene, encoding the Myosin9 subunit of Myosin2A, which includes May-Hegglin anomaly, Sebastian syndrome, Fechtner syndrome, and Epstein syndrome. Three forms of Myosin2 (Myosin 2A, Myosin2B and Myosin2C) exist and perform several function in various cell types usually in combinations. Yet, certain blood cells, including platelets and leukocytes, only express Myosin2A explaining the bleeding problems and immune abnormalities observed in MYH9-RD patients. More than 45 mutations in MYH9 gene have been associated to MYH9-RDs. The severity of symptoms varies with mutations inside the motor domain leading to more severe disorders than mutations in the tail-domain (Althaus and Greinacher, 2009; Sanborn et al., 2011). For example, mutations in the Myosin9 head- and N-terminal S2-domain (Ser96Leu and Thr1155Ile) strongly affected NK-cell cytotoxicity, whereas mutations more C-terminal (Arg1400Trp and Asp1424Asn) yielded milder effects. However, NK-cell cytotoxicity of a patient with a 5779delC mutation (leading to a Myosin9 truncation at residue 1942) showed again strongly diminished NK-cell cytotoxicity. This truncation lacks an important phosphorylation site essential for the interaction between Myosin2A and LGs leading to defective LGs transport (Sanborn et al., 2011). Myosin motors control the actin based step in intracellular transport of LE/Lys and LROs. Mutations then result in various genetic diseases. Also these actin-based motor proteins need to find the correct vesicles by interacting with defined adaptors, which can also be subject to mutations and disease phenotypes.

## Motor adaptors at the lysosomal surface and diseases

The LE/Lys outer membrane is decorated with proteins regulating LE/Lys trafficking and functions including protein sorting, fusion with other organelles and protein degradation. The small GTPase Rab7 marks these organelles and controls HOmotypic fusion and vacuole Protein Sorting (HOPS) complex and motor protein recruitment (Jordens et al., 2001; Johansson et al., 2007; Pankiv et al., 2010; van der Kant et al., 2013; Raiborg et al., 2015). Defects in these proteins are mainly associated to neurodegenerative diseases. This includes defects in the retromer-complex associated to Parkinsons disease (Harrington et al., 2012; Zimprich, 2011; Vilarino-Guell et al., 2011), mutations in HOPS-complex subunits associated to Schizophrenia (Xu et al., 2012), Rab7 mutations linked to Alzheimers disease and CMT2 (Romano et al., 2022; Meggouh et al., 2006; Vardarajan et al., 2012; Verhoeven et al., 2003; Houlden et al., 2004; Wang et al., 2014) and VAPB mutations involved in ALS development (Vardarajan et al., 2012). These neurological problems are due to the absence of substrate degrading enzymes resulting in LE/Lys misfunctioning and accumulation of undigested and accumulated material inside the neuronal cell. For example, Alzheimers disease is described to accumulate Tau-containing neurofilaments and Beta-amyloid plaques formed from APP (Amyloid Precursor Protein) (Grundke-Iqbal et al., 1986; Glenner and Wong, 1984; Delacourte and Defossez, 1986; Kosik et al., 1986; Wood et al., 1986; Nukina and Ihara, 1986; Kang et al., 1987). In addition, missense mutations affecting Rab7a, the GTPase important for both dynein-dynactin and kinesin-mediated LE/Lys transport, leads to decreased presence of autolysosomes suggesting limited clearance of intracellular protein aggregates which may explain its association to CMT2 (Romano et al., 2022; Meggouh et al., 2006; Verhoeven et al., 2003; Houlden et al., 2004; Wang et al., 2014). Also, mutations in the proteins CLN3 (leading to a truncated protein) and CLN5 (leading to a misfolded protein) disturb LE/Lys trafficking and sorting machineries. CLN3 is important for recruiting Rab7 and failure affects Rab7-associated functions including motor recruitment, whereas CLN5 interacts with the retromer complex for protein sorting. Insufficient recruitment of either the motor proteins or sorting complexes leads to the accumulation of ceroid lipofuscin and consequently the neurodegenerative disorder Neuronal Ceroid Lipofuscinois (Uusi-Rauva et al., 2012; Mamo et al., 2012). Another group of LE/Lys related diseases are Lysosomal storage disorders (LSDs), including Niemann Pick disease Type-C, also resulting from accumulated undigested material inside the lysosome. Gene mutations associated to LSDs are mutations in *NPC1* and *NPC2* (mostly single amino acid mutations reducing or eliminating their cholesterol transport activities), resulting in accumulation of cholesterol in LE/Lys, which is sensed by the cholesterol-sensor ORP1L (Rocha et al., 2009; Chang et al., 2005). As a result, ORP1L fails to remove the dynein-dynactin motor from Rab7-RILP resulting in net minus-end transport and accumulation of LE/Lys close to the nucleus around the centriole/MTOC (Rocha et al., 2009). Also, mutations in the effector proteins for Rab7 and other LE/Lys GTPases may result in disease. Indeed, mutations in the Rab7 effector FYCO1 are associated with Cataract (Barashkov et al., 2021; Ullah et al., 2023; Aprahamian et al., 2021; Chen et al., 2011; Iqbal et al., 2020; Mei et al., 2022; Saleem et al., 2022; Shirzadeh et al., 2022).

The dual-adaptor protein huntingtin is mutated in Huntington’s disease (Marcy et al., 1993). Wild-type huntingtin (no capital) is involved in various cellular processes related to not only endosomal transport but also transcriptional regulation and synaptic functioning (Barron et al., 2021; Benn et al., 2008; Dunah et al., 2002). Huntingtin may act as a switch between dynein-dynactin and kinesin-mediated LE/Lys transport. Via the huntingtin-interacting protein HAP1 (Harjes and Wanker, 2003; Caviston and Holzbaur, 2009; Li and Li, 2004; Truant et al., 2006), both the kinesin-1 and dynein/dynactin motor complex can be recruited to the same LE/Lys vesicle (Twelvetrees et al., 2010; Caviston et al., 2007; McGuire et al., 2006; Engelender et al., 1997; Li et al., 1998). Depending on its phosphorylation status, huntingtin can regulate switches in transport direction. Whereas kinesin-1 interacts with phosphorylated huntingtin for plus-end directed transport, kinesin-1 is removed upon huntingtin dephosphorylation thereby favoring dynein-mediated transport in the opposite direction (Colin et al., 2008). In Huntington’s disease patients, the huntingtin gene *Htt* contains a prolonged CAG repeat (Gil and Rego, 2008). Because huntingtin performs multiple roles in the cell, this mutation is suggested to have multiple pathogenic mechanism, including aggregate formation, altered gene expression, mitochondrial dysfunction and impaired autophagy (reviewed in (Jimenez-Sanchez et al., 2017; Tong et al., 2024)). Focusing on the effect on endosomal transport, the expanded CAG repeat was found to limit transport of BDNF-containing neuronal vesicles by affecting dynein/dynactin motor formation as well as disruption of their association to microtubules (Gauthier et al., 2004).

It is likely that other transport-associated proteins whose function cannot be easily compensated also cause diseases in neurological, immunological or other systems relying on proper LE/Lys or LRO transport. Indeed, novel disease causing mutations in the vesicle transport machinery are still being identified. The functioning of LROs similarly depends on trafficking factors. How do mutations in proteins specifically involved in LRO dynamics associate to different diseases?

### The motor adaptors at the LRO membrane and diseases

Although LROs share many transport characteristics with LE/Lys, their specialized character provides them with their own set of transport-related membrane proteins. Consequently, when these proteins are dysfunctional, specialized cargo secretion will be affected only in these specialized cells. For example, transport related defects in melanocytes results in pigmentation defects resulting in albinism. This is observed in Griscelli syndrome (GS) two and 3, Chediak-Higashi syndrome (CHS) and Hermansky-Pudlak syndrome (HPS) (Dell'Angelica et al., 2000). GS2 and GS3 are caused by mutations in the *RAB27A* gene or *MLPH* gene (encoding for the Rab27a effector Melanophilin), respectively. While GS2 and GS3 patients both show pigmentation defects due to abnormal melanin secretion, only GS2 patients have additional immune defects, such as hemophagocytic lymphohistiocystosis. This difference in symptoms is caused by the unique function of melanophilin in melanosome secretion as part of the Rab27A-Melanophilin-Myosin5 complex, whereas Rab27A also mediates LGs transport in CTLs as part of the RAB27A-Slp3a-kinesin-1 (KIF5B) complex (Kurowska et al., 2012). Several mutations in the LYST gene, encoding for LYSosomal Trafficking regulator, have been identified in CHS patients (Karim et al., 2002; Dufourcq-Lagelouse et al., 1999; Barbosa et al., 1997; Barbosa et al., 1996; Karim et al., 1997; Morimoto et al., 2024). Although the function of LYST is not exactly known, cells of these patients expressing a short truncated LYST version show an increased LE/Lys and LRO size resulting in an altered structure and function (Ji et al., 2016). Enlarged melanosomes containing accumulated melanin are found in these patients as well as enlarged platelet dense bodies resulting in defective platelet-function and neurological abnormalities (Ji et al., 2016). Another melanosome-associated gene mutated in HPS is the *HPS1* gene, encoding for HPS1, a subunit of the Biogenesis of Lysosomal-related Organelles Complex (BLOC)-3 complex. BLOC-3 is a GEF for Rab32 and Rab38. Mutated HPS1 fails to activate the GTPases Rab32 and Rab38, then inhibiting melanin release (Gerondopoulos et al., 2012). Rab32 defects are also related to Parkinson Disease (Gustavsson et al., 2024; Hop et al., 2024). As described above, some LRO membrane proteins have specific roles in controlling their intracellular transport in certain cell types, while others fulfill general functions in LRO and LE/Lys transport. When mutated, they affect one, two or multiple cell types. Different combinations of neurological defects, immune and blood deficiencies as well as pigmentation abnormalities are therefore often seen in patients with mutations in these LRO transport regulating genes.

## Conclusion

**FIGURE 2:**
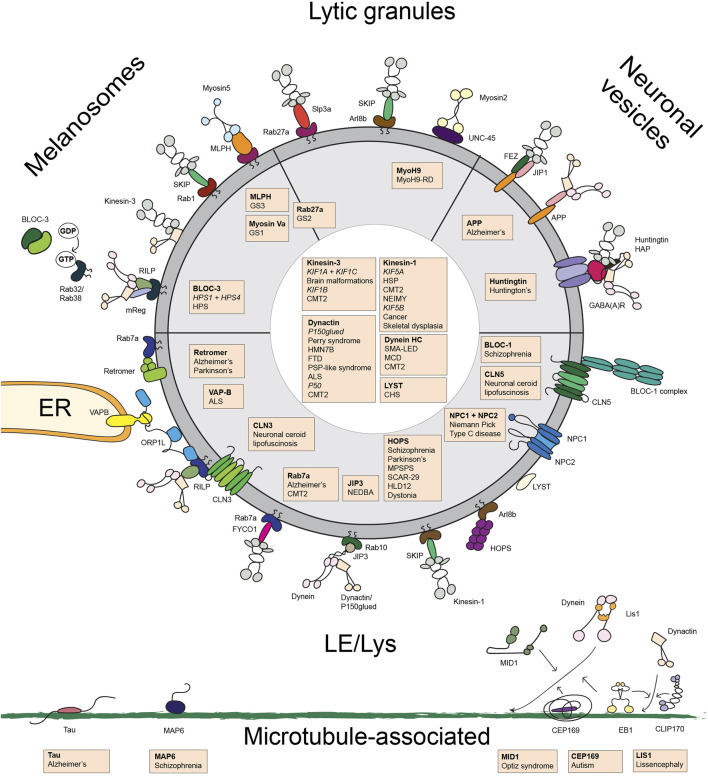
Overview of the transport machinery controlling the transport of late endosomes/lysosomes, melanosomes, lytic granules and and neuronal vesicles. Schematic overview of the proteins involved in late endosome/lysosome and lysosome-related organelle transport. Proteins of which mutations are associated with disease are indicated in orange boxes with a list of the associated disease. Abbreviations used: ALS, amyotrophic lateral sclerosis; BLOC, Biogenesis of Lysosomal-related Organelles Complex; CHS, Chediak-Higashi syndrome; CMT2, Charcot Marie Tooth disease type 2; FTD, frontotemporal dementia; GS, Griscelli syndrome; HLD12, Hypomyelinating Leukodystrophy 12; HMN7B, hereditary motor neuronopathy 7B; HOPS, HOmotypic fusion and vacuole Protein Sorting; HSD hereditary spastic paraplegia; HSP, Hereditary Spastic Paraplegia; MCD, malformations in cortical development; MLPH, melanophilin; MPSPS, mucopolysaccharidosis-plus syndrome; MyoH9-RD, myosinH9-related disease; NEDBA, neurodevelopmental disorder with or without variable brain abnormalities; NEIMY, Neonatal Intractable Myoclonus; NPC, Niemann Pick Type C; SCAR-29, autosomal recessive spinocerebellar ataxia-29; SMALED, spinal muscular atrophy with lower extremity dominance.

The transport of LE/Lys and LROs involves many large and small intracellular structures that in assembly coordinate proper cargo transport (Figure 2). Its importance is best illustrated by the fact that many proteins involved in this process cause neurological or other disorders when mutated. These mutations can occur at the level of the highways (microtubules or actin), the signboards on these highways (post-translational mutations, MAPs), lorries (the motor proteins) and their cargo (the adaptor/effector proteins linking motor proteins to their cargo). We begin to understand the system and the reason for the associated diseases. The next challenge will be ways to correct the traffic jams and often lethal accidents.

## Abbreviations

ALS, amyotrophic lateral sclerosis; APP, β-amyloid precursor protein; Arl, Arf-like; Arp1, actin-related protein-1; ASD, autism spectrum disorder; BLOC, Biogenesis of Lysosomal-related Organelles Complex; CMT2, Charcot Marie Tooth disease type 2; CSCSC2, congenital symmetric circumferential skin creases-2; CTL, cytotoxic T cell; HC, heavy chain; HMN7B, hereditary motor neuronopathy 7B; HPS, Hermansky-Pudlak syndrome; HOPS, HOmotypic fusesion and vacuole Protein Sorting; HSD, hereditary spastic paraplegia; IC, intermediate chain; KAP3, kinesin associated protein 3; LC, light chain; LE/Lys, late endosome/lysosome; LG, lytic granule; LIC, light intermediate chain; LRO, lysosome related organelle; MAP, microtubule-associated protein; MCD, malformations in cortical development; MTOC, microtubule-organizing center; MYH9, Myosin9; NEDBA, neurodevelopmental disorder with or without variable brain abnormalities; NK-cell, natural killer cell; PP2A, phosphatase 2A; SMALED, spinal muscular atrophy with lower extremity dominance; TDP-43, transactive-response DNA-binding protein of 43 kDa.
